# The MET Oncogene: An Update on Targeting Strategies

**DOI:** 10.3390/ph17111473

**Published:** 2024-11-02

**Authors:** Simona Gallo, Consolata Beatrice Folco, Tiziana Crepaldi

**Affiliations:** 1 Department of Oncology, University of Turin, Regione Gonzole 10, 10143 Orbassano, Italy; simona.gallo@unito.it (S.G.); consolatabeatrice.folco@unito.it (C.B.F.); 2 Candiolo Cancer Institute, FPO-IRCCS, SP142, Km 3.95, 10060 Candiolo, Italy

**Keywords:** HGF/MET, cancer, drugs

## Abstract

The MET receptor, commonly known as HGF (hepatocyte growth factor) receptor, is a focus of extensive scientific research. MET has been linked to embryonic development, tissue regeneration following injury, tumorigenesis, and cancer metastasis. These functions underscore its involvement in numerous cellular processes, including stemness, proliferation, motility, cell dissociation, and survival. However, the enigmatic nature of MET becomes apparent in the context of cancer. When MET remains persistently activated, since its gene undergoes genetic alterations, it initiates a complex signaling cascade setting in motion an aggressive and metastatic program that is characteristic of malignant cells and is known as “invasive growth”. The expanding knowledge of MET signaling has opened up numerous opportunities for therapeutic interventions, particularly in the realm of oncology. Targeting MET presents a promising strategy for developing novel anti-cancer treatments. In this review, we provide an updated overview of drugs designed to modulate MET signaling, highlighting MET kinase inhibitors, degraders, anti-MET/HGF monoclonal antibodies, and MET-targeted antibody–drug conjugates. Through this review, we aim to contribute to the ongoing advancement of therapeutic strategies targeting MET signaling.

## 1. Introduction

**Figure 1:**
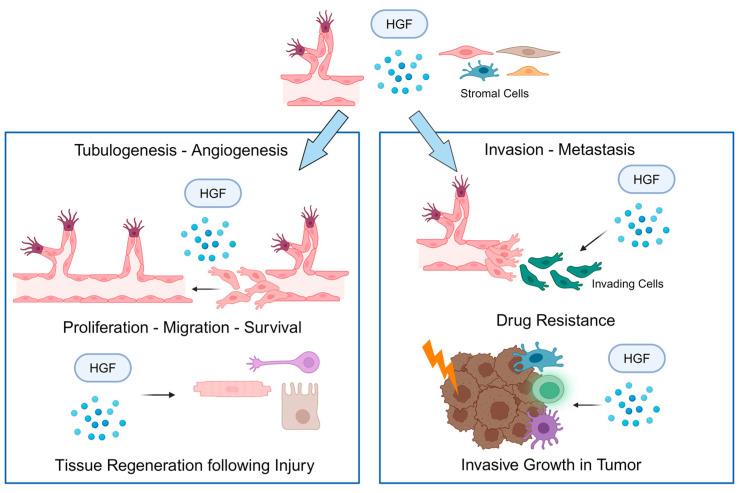
MET biological functions in physiological contexts and during tumorigenesis. The HGF/MET axis sustains various morphogenetic processes, including tubulogenesis and angiogenesis, as well as proliferation, migration, and survival, during tissue regeneration following injury. When MET undergoes abnormal activation, it acts as oncogenic driver, contributing to cancer cell proliferation, survival, invasion, metastasis, and drug resistance. This program, induced by the HGF/MET axis, is known as “invasive growth”. Created in BioRender. Crepaldi, T. Available online: https://app.biorender.com/illustrations/66f11f91af9262b7665ac0ce (accessed on 31 October 2024). BioRender(accessed on 31 October 2024).

MET is a receptor tyrosine kinase (RTK) activated by its cognate ligand, the hepatocyte growth factor (HGF). HGF, originally discovered as a potent mitogen for hepatocytes [1], was later identified as a stromal factor promoting cell motility, also known as scatter factor (SF) [2]. The HGF/MET axis sustains various cellular processes, including proliferation, migration, and survival. These processes are critical during embryonic development—such as in epithelial-to-mesenchymal transition (EMT) and epithelial tubulogenesis—as well as in postnatal and adult activities, including angiogenesis and tissue regeneration following injury (Figure 1) [3,4]. The *MET* gene is primarily expressed in epithelial and endothelial cells [4], and it is also expressed in stem/progenitor cells such as myoblasts [5] and neuronal precursors [6], contributing to the development of various tissue structures [7,8,9,10]. Indeed, the functions of HGF and MET are pivotal in embryonic development of neuroectodermal tissues, which are shaped through high rates of migration and survival during their route. In mice lacking *Hgf* or *Met* genes, there is a significant disruption in liver development, and the placental labyrinth trophoblasts exhibit reduced growth, resulting in prenatal mortality [7,8,11]. The deletion of these genes also results in the total absence of hypaxial muscles, including those in the diaphragm, limbs, and tongue [5].

In tubulogenesis and angiogenesis, HGF promotes proliferation and migration of epithelial and endothelial cells, respectively (Figure 1) [12,13,14]. Initially, a partial EMT occurs, wherein densely packed cells adopt a spindle-shaped morphology, releasing cytoplasmic extensions into the surrounding matrix. These extensions grow into single-file chains that resist an apoptotic process called “anoikis” to lengthen and thicken. Ultimately, the cells redifferentiate, regaining polarity and forming solid cords that mature into tubules through the gradual development of a continuous lumen. At the signaling level, the sequence of events necessary for HGF-dependent tubule formation depends on the stability of the STAT3 and NF-κB pathways [15,16]. HGF also induces strong proliferative and anti-apoptotic responses in renal epithelial cells by activating Ras-dependent mitogenic signals, the PI3K/Akt pro-survival pathway, and anti-apoptotic effectors such as Bcl-xL and Bcl-2. This potent reno-protective action promotes kidney regeneration and prevents acute renal failure induced by tubular necrosis [17]. Adult wound healing and tissue regeneration involve a similar mechanism, where residual cells migrate into injured locations to reconstruct pre-existing structures [18]. During wound healing, the hyperproliferative epithelium, composed of marginal keratinocytes at the wound borders, divides to produce new cells that move over the injured dermis matrix and repopulate the wound area [19,20]. HGF is required in vitro for the closure of scratch wounds, helping to reorient keratinocytes. This process ensures that the plus ends of microtubules, actin stress fibers, and focal adhesion components point toward the wound edges, facilitating cell motility. Key signaling effectors in this process include Gab1, Akt, ERK1, ERK2, and p21-activated kinases 1 and 2 (PAK1 and PAK2), which are targets of RhoA and regulate actin polymerization and protrusion formation [20].

## 2. The Dark Side of MET

The MET receptor tyrosine kinase is usually kept at low activity levels in normal, healthy cells. However, when abnormally activated, it becomes a recognized oncogenic driver [21]. MET initiates a complex network of signaling cascades that reprogram gene expression, driving the HGF/MET-induced “invasive growth” program, characterized by enhanced cell motility, resistance to apoptosis, proliferation, long-distance tissue migration, and resilience to various stressors [22,23]. Moreover, the MET’s ability to activate this invasive growth program is closely linked to its efficiency in mitigating unfavorable microenvironmental conditions [24]. Tumor invasiveness exploits molecular mechanisms typically involved in embryogenesis and tissue regeneration (Figure 1). Cancer cells activate cellular rearrangement schemes usually limited to developmental and regenerative processes, promoting metastasis [25]. The concept of invasive growth encompasses EMT, a reversible state that involves both cell stemness and dissemination. This process enables cancer cells to migrate over long distances while preserving their aggressive phenotype and surviving under stress [26]. Persistent activation of the Raf–Mek–ERK pathway is essential for the early stages of EMT [27]. Invasive epithelial cells avoid “anoikis” by resisting apoptosis, a key feature of EMT that allows the cells to survive without the usual support from cell–cell and cell–matrix interactions. MET activation stimulates the PI3K/Akt pathway, which protects the cells from death [28].

In a limited number of tumors, the *MET* gene undergoes genetic alterations—mostly amplifications and/or point mutations—resulting in kinase hyperactivation, which is necessary to sustain the transformed phenotype, also known as “oncogene addiction” [21,29,30,31]. *MET* gene amplification, which leads to a constitutively active receptor due to overexpression, has been identified in patients with gastric cancers, lung tumors, renal cell carcinomas, hepatocellular carcinomas, ovarian tumors, melanomas, and triple-negative breast cancer (TNBC) [21]. Moreover, *MET* gene amplification has been recognized as a mechanism of resistance to EGFR inhibitors in metastatic colorectal cancer and non-small-cell lung cancer (NSCLC) [32,33,34]. Point mutations affecting both the catalytic and regulatory domains, causing constitutive receptor kinase activity, have been found in hereditary papillary renal cancer, lymphomas, carcinomas, and aggressive breast tumors [21,35,36]. A clinically relevant phenomenon involves mutations in the splicing sites flanking MET exon 14, leading to deletion of regulatory sequences involved in receptor degradation [21]. This genetic alteration is linked to various types of lung cancers, including lung adenocarcinomas (approximately 3%), lung squamous carcinomas (around 5%), and a substantial percentage of highly aggressive and chemoresistant lung sarcomatoid tumors (20-30%), a rare but notable subset (constituting approximately 1% of all lung malignancies) [37,38,39,40]. Chromosomal rearrangements involving the *MET* gene also play a role in tumorigenesis. The translocated promoter region *(TPR)-MET*, a transforming oncogene generated by chromosomal rearrangement, has been found in precursor lesions of human stomach malignancies, suggesting a genetic predisposition to gastric carcinoma formation due to this translocation [41]. The oncogenic TPR-MET fusion protein is constitutively active, due to a leucine-zipper domain within the TPR moiety, which provides a structural dimerization motif that maintains the active enzyme. In murine models, transgenic expression of TPR-MET driven by a ubiquitous promoter results in the development of breast tumors and other cancers [42]. Moreover, the fusion of the *MET* sequence at its 5′ end with *PTPRZ1* gene exons creates the *PTPRZ1-MET* fusion, which has been reported in brain cancers, including secondary glioblastomas and low-grade gliomas [43]. MET’s role in sustaining cell adaptation to unfavorable environmental conditions—a feature known as “oncogene expedience”—is crucial in metastasis [30]. In many cases, MET activation intensifies the malignancy of already transformed cells, a secondary event often produced by transcriptional up-regulation from other oncogenes (e.g., Ras) [44], environmental conditions (like hypoxia) [45], or molecules secreted by the reactive stroma (e.g., pro-angiogenic factors, inflammatory cytokines, and HGF) [46,47]. The role of MET signaling in tumors has been partially elucidated through the modification of consensus sequences that enable the recruitment of unique transducers. Ras signals are primarily involved in MET-induced cell proliferation, whereas PI3K recruitment is necessary for the induction of cell motility [48]. Despite the different mechanisms, *MET* alterations share a common role in promoting invasive growth, making tumor cells vulnerable to targeted therapies. Several MET-targeting agents, including MET antibodies and small-molecule kinase inhibitors, are currently envisaged as anti-cancer therapeutics [21,37,49].

## 3. The Negative Regulation of MET Activity

**Figure 2:**
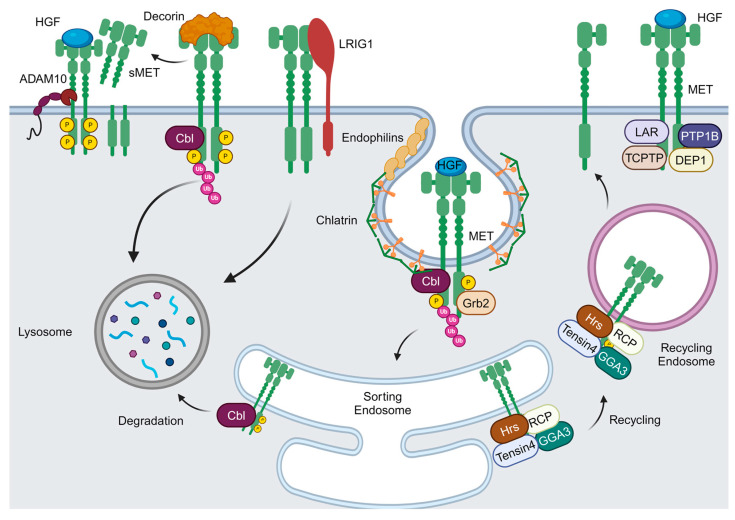
MET negative regulation by internalization, degradation, and recycling. Several mechanisms negatively regulate MET activity. Upon HGF activation, MET is degraded by the proteasome after interaction with Cbl, an ubiquitinase enzyme, Grb2, and endophilins. Alternatively, MET can be recycled back to the plasma membrane through interaction in the sorting endosome with Hrs, GGA3, Tensin-4, and RCP. MET degradation can also occur through ligand-independent mechanisms: LRIG1 induces Cbl-independent MET degradation, and Decorin stimulates transient activation of MET, promoting its internalization/degradation and ectodomain shedding. This shedding results in the release of sMET, a soluble decoy form of MET, which is produced by metalloproteases, such as ADAM10. Finally, MET activity is further modulated by phosphatases, including PTP1B, TCPTP, DEP1, and LAR. Created in BioRender. Crepaldi, T. Available online: https://app.biorender.com/illustrations/66fbbab47baf997aec37d1ed (accessed on 31 October 2024). Created in BioRender. Crepaldi, T. (2024) BioRender.com/q46r260 (31 October 2024).

The biological response mediated by MET depends on the receptor’s localization and signalosome maintenance over time. Several mechanisms negatively regulate MET activity, such as its rapid internalization, degradation, and recycling (Figure 2) [50].

After HGF stimulation, MET is polyubiquitinated and targeted for proteasomal degradation through an internalization process involving interactions with various molecular mediators, including Grb2, endophilins, and Cbl [50,51,52]. Ubiquitination is facilitated by Cbl, which is recruited to the juxtamembrane domain (Y1003) of the MET receptor. Both the ligand-activated MET receptor and its immediate downstream signaling partners are targeted for degradation. Oncogenic mutations in this domain can impair receptor degradation, leading to prolonged signaling and tumorigenesis [53]. Interestingly, while Cbl is mainly involved in receptor degradation, it is not required for internalization [52]. Internalized MET is not exclusively destined for degradation, but it can be recycled back to the plasma membrane via a process involving the sorting endosome and different MET interactors: Hrs, Golgi-localized γ-ear-containing Arf-binding protein 3 (GGA3), Tensin-4, and Rab coupling protein (RCP) [54,55,56,57]. MET mutations can increase receptor recycling, resulting in aberrant downstream signaling that induces the invasive growth program in renal carcinoma [58].

Various ligand-independent mechanisms for the degradation of membrane-anchored MET have been identified [59]. In particular, immunoglobulin-like domain-containing protein 1 (LRIG1) interacts with MET, inducing its down-regulation through the lysosomal compartment without the involvement of Cbl [59]. Moreover, Decorin, a member of the small leucine-rich proteoglycan family, interacts with MET, inducing its tyrosine phosphorylation, which, while unable of initiating biological responses, promotes efficient MET down-regulation by Cbl recruitment [59]. Interestingly, Decorin also appears to induce MET degradation through ectodomain shedding [59]. MET shedding by metalloproteases, such as ADAM10, represents an additional mechanism for receptor down-regulation. Specifically, ADAM10 cleavage of MET results in the receptor degradation and release of soluble MET (sMET) in the extracellular space [60,61]. Finally, MET activity is further modulated by phosphatases such as PTP1B, TCPTP, DEP1, and LAR [62]. Receptor localization is critical for signal transduction. For instance, the spatial positioning of activated MET dictates its signaling outcomes: perinuclear localization facilitates MET-dependent STAT3 activation [63], while Rac1 regulation differs depending on whether MET is in peripheral or perinuclear endosomes localization [64].

## 4. Drugs Targeting MET

**Figure 3:**
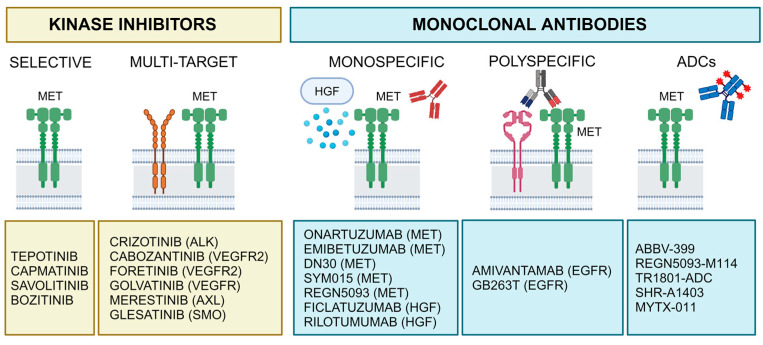
MET targeting strategies. Numerous strategies have been developed to target MET. MET kinase inhibitors selectively target MET or multiple RTKs, monoclonal antibodies specifically target MET or HGF, polyspecific antibodies target multiple RTKs, and MET-targeted antibody-drug conjugates (ADCs) combine the specificity of MET monoclonal antibodies with the cytotoxic potency of chemotherapeutic agents. Created in BioRender. Crepaldi, T. Available online: https://app.biorender.com/illustrations/66f132447cc06bb44506f426 (accessed on 31 October 2024). Created in BioRender. Crepaldi, T. (2024) BioRender.com/e40 × 012 (31 October 2024).

The growing understanding of MET signaling has created numerous opportunities for therapeutic interventions, especially in oncology. Targeting MET presents a promising strategy for developing novel anti-cancer treatments. Below, we explore several key applications for drugs designed to modulate MET signaling (Figure 3 and Table 1).

### 4.1. MET Kinase Inhibitors

**Figure 4:**
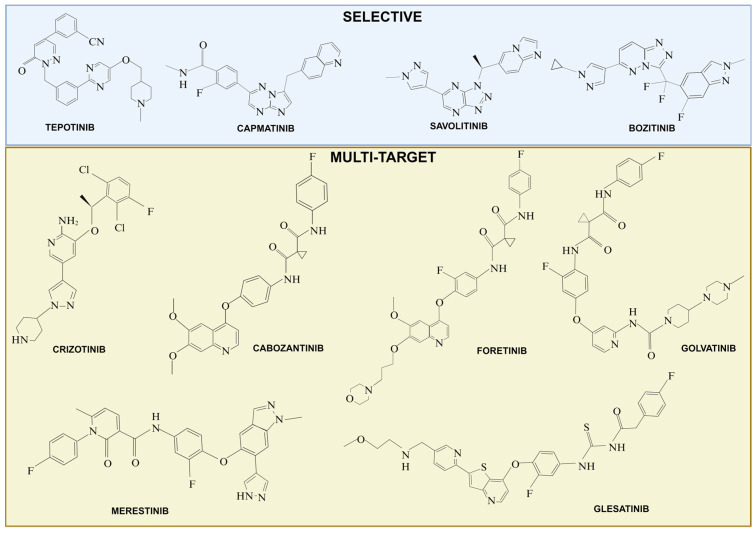
Chemical structures of MET-targeting kinase inhibitors. Created in BioRender. Crepaldi, T. Available online: https://app.biorender.com/illustrations/671b778a541cebcc1822d471 (accessed on 31st October 2024). Created in BioRender. Crepaldi, T. (2024) https://BioRender.com/j38q128 (accessed on date).

Several drug development strategies focus on MET signaling modulation, such as small-molecule kinase inhibitors, and monoclonal antibodies targeting MET or HGF. There are many excellent reviews on the use of MET-targeting agents in the clinical setting [21,90,134,135,136]. Recent studies have selected biomarkers and thresholds for MET-inhibitor trials. This approach has contributed to the success of MET TKIs like crizotinib, tepotinib, capmatinib, and savolitinib (Figure 4). A significant milestone in identifying the right biomarkers for MET inhibitors was the discovery of MET exon 14 skipping mutations, which marked a key breakthrough in the field [65,136]. Tepotinib, capmatinib, and savolitinib have been specifically designed and optimized to target MET exon 14 skipping mutations, showing efficacy in NSCLC with these mutations [66,69,70,71,76]. Several case reports have documented remarkable responses to MET-targeted therapies in patients with MET-driven cancers. One case involved a patient with MET-amplified biliary tract cancer, demonstrating a long-term response to tepotinib [77]. This outcome is particularly noteworthy because MET amplification often indicates an aggressive cancer phenotype, yet tepotinib helped manage tumor progression effectively in this case. A patient with intrahepatic cholangiocarcinoma, a rare liver cancer type, showed a significant response to capmatinib [78]. This patient’s tumor harbored a unique *TFG-MET* gene fusion, which is relatively uncommon and may have contributed to the favorable response. The treatment led to tumor shrinkage and manageable side effects, highlighting the potential of capmatinib in patients with specific genetic alterations like MET fusions. In a case report by Li et al. [79], a patient with lung adenocarcinoma harboring a MET exon 14 skipping mutation exhibited a remarkable response after receiving neoadjuvant therapy with tepotinib. Notably, capmatinib demonstrated a profound intracranial response in a crizotinib-resistant brain lesion of an advanced NSCLC patient with the same MET exon 14 skipping mutation, suggesting superior central nervous system penetration and effectiveness in treating brain metastases [80]. This makes capmatinib a promising option for CNS-metastasized NSCLC patients with this specific MET mutation. Moreover, a case report by Tian et al. [84] highlighted a dramatic response in a patient with marginally resectable lung adenocarcinoma containing a MET exon 14 skipping mutation following neoadjuvant treatment with savolitinib. Significant tumor shrinkage enabled successful surgical resection, previously considered challenging. This report underscores the potential of savolitinib in improving surgical outcomes in patients with specific genetic alterations. Another highly selective MET inhibitor, bozitinib (also known as PLB-1001 or vebreltinib, Figure 4), exhibits blood–brain barrier permeability and has shown effective inhibition of MET-driven glioma progression in both cell lines and xenograft models [85]. Moreover, bozitinib showed efficacy in NSCLC patients with MET exon 14 skipping mutations [86]. A case report by Huang et al. [87] describes a patient with NSCLC who, after failing initial treatment with tepotinib, achieved a prolonged response with vebreltinib, a highly selective MET inhibitor. This success suggests that sequencing or switching MET inhibitors may benefit patients with MET-driven cancers who develop resistance to initial therapies. Crizotinib, originally developed as an ALK inhibitor, also targets MET in NSCLC with MET amplifications or MET exon 14 alterations [88,91,92]. The development and approval of these drugs (Table 1) underscore the importance of molecular-driven approaches in oncology, where specific genetic alterations guide treatment decisions. Cabozantinib targets MET along with VEGFR2, making it effective in treating several metastatic cancers, including metastatic colorectal cancer (Figure 4) [81]. In fact, targeting MET alongside VEGF inhibition can reduce tumor aggressiveness in preclinical pancreatic and neuroblastoma cancers [72,73]. Cabozantinib is also used in patients’ thyroid and renal cancers [67,74]. Further optimization of these anti-MET TKI molecular structures has been performed to improve the druglike properties [81]. Other multi-target MET TKIs that target both MET and an additional RTK (e.g., foretinib, golvatinib, merestinib, glesatinib; Figure 4) have been developed in recent years and are currently under clinical investigation. These drugs can greatly enhance efficacy and address some of the limitations associated with single-target inhibitors, such as drug resistance [137]. Several clinical reports described secondary MET mutations as mechanisms for crizotinib resistance. Interestingly, one case of a patient with advanced lung cancer with a MET exon 14 skipping mutation and D1246N was reported during treatment with crizotinib [101]. For more detailed examinations of clinical trials, there are specialized reviews on this subject [90,102]. Molecular stratification of patients with genetically altered *MET* tumors has improved the identification of those who are likely to respond to treatment. Tumor types that are expected to benefit most from MET-targeted therapy are primarily found in lung cancer, papillary renal carcinoma, digestive system tumors, and brain gliomas [88,103,105,106,107,108,138].

### 4.2. Emerging Strategies in MET-Targeted Protein Degradation

New strategies targeting MET through a small-molecule degrader approach have been proposed [109,110,111,112,139]. This approach, called targeted protein degradation (TPD), focuses on using small molecules to selectively eliminate specific proteins within cells, rather than merely inhibiting their activity. It shows promising potential in cancer treatment, as it reduces off-target effects and addresses drug resistance issues. The most used TPD technology is use of proteolysis-targeting chimeras (PROTACs), bifunctional molecules consisting of a targeting moiety that binds the target of interest and an E3 ligase recruiter, facilitating proteasomal degradation [113]. Recent studies have demonstrated the efficacy of MET-targeting PROTACs in reducing tumor growth, highlighting their potential as a novel therapeutic approach in various malignancies, particularly those driven by aberrant MET signaling [109,110,111,112]. Other TPD strategies are also emerging, including hydrophobic tags (HyT) [114]. The HyT technology links small-molecule inhibitors with hydrophobic groups that bind to the protein surface, inducing misfolding and subsequent proteasomal degradation [114]. Min et al. [139] synthesized a novel MET HyT TPD inhibitor that demonstrated potent anti-tumor activity in hepatocellular carcinoma. Challenges remain in optimizing delivery and mitigating off-target effects, but ongoing research aims to improve the clinical applicability of this innovative strategy.

### 4.3. MET-Targeted Monoclonal Antibodies

Onartuzumab is a one-armed monoclonal antibody that binds the extracellular domain of the MET receptor and blocks its interaction with HGF. In the initial onartuzumab trials, MET protein overexpression was the only criterion for patient enrollment, due to its high prevalence in NSCLCs and its association with poor prognosis [115,116]. A combination of onartuzumab with standard first-line chemotherapy did not significantly improve clinical benefits to patients with gastroesophageal carcinoma [117]. The failure of clinical trials with onartuzumab may be due to either the lack of molecular stratification in patients or inadequate inhibition of activated MET receptors due to ligand-independent mechanisms, such as specific mutations and gene amplifications [118].

Emibetuzumab (LY2875358) is a humanized bivalent monoclonal antibody that targets both ligand-dependent and ligand-independent MET signaling by blocking HGF binding to MET and promoting its internalization and degradation [120]. A phase II study in metastatic NSCLC patients with EGFR mutations found no significant difference in median progression-free survival between those receiving emibetuzumab plus erlotinib and those receiving erlotinib alone [140]. However, high MET expression emerged as a negative prognostic marker, suggesting that emibetuzumab combined with erlotinib might offer clinical benefits for patients with high MET expression.

A potential therapeutic strategy involves inducing MET intramembrane proteolysis in cancer cells that overexpress the MET receptor. This approach decreases MET levels and generates sMET, which may help mitigate signaling pathways driving tumor growth. The DN30 monoclonal antibody can promote this process [121,122]. DN30, which targets the extracellular domain of MET, induces the cleavage of MET by ADAM10, leading to receptor degradation and the production of sMET in the extracellular space along with unstable fragments inside the cell [124]. The DN30 antibody has demonstrated effectiveness in reducing tumor growth in animal models, particularly in tumors with constitutive MET activation due to gene amplification. Research efforts have been focused on improving DN30’s efficacy by developing a monovalent version to prevent MET dimerization and enhancing its stability through molecular and chemical modifications [125]. Additionally, an innovative approach has combined DN30 with a modified sMET decoy that lacks the ability to bind DN30, due to a point mutation in its binding domain, further refining its therapeutic potential [126].

SYM015 is a combination of two humanized IgG1 monoclonal antibodies (Hu9006 and Hu9338) that target distinct, non-overlapping epitopes within the SEMA (semaphoring-like domain) α-domain of MET. This antibody duo exhibits strong activity both in vitro and in vivo in models with MET amplification and MET exon 14 mutations and is currently undergoing clinical development [127,128].

REGN5093 is a biparatopic METxMET antibody, with each arm recognizing a separate epitope within the MET SEMA domain [129]. It exhibits greater efficacy than what would be expected based on the characteristics of the original antibodies. It promotes MET internalization and inhibits its recycling, with the effect on recycling being the key factor that differentiates the biparatopic antibody from the original antibodies.

Amivantamab (JNJ-61186372) is a bispecific antibody that targets both EGFR and MET, demonstrating preclinical efficacy in EGFR-mutated NSCLC models and showing promise in a first-in-human study for advanced NSCLC [130]. Amivantamab operates through several mechanisms, as demonstrated in preclinical models. These include blocking ligand binding; promoting receptor endocytosis and degradation; and engaging macrophages, monocytes, and natural killer cells via its Fc domain [131]. Its effectiveness in tumors with Exon20 insertions (Exon20ins) has also been reported [132]. Studies using Ba/F3 cells, patient-derived cells, organoids, and xenografts with various Exon20ins demonstrated that amivantamab inhibits tumor growth by reducing EGFR-MET levels and enhancing immune response, indicated by increased IFNγ secretion. Amivantamab has been approved for treating patients with advanced NSCLC harboring EGFR exon 20 insertions based on results from the phase I CHRYSALIS trial [133]. The trial demonstrated a 40% objective response rate and a median response duration of 11.1 months. Patients had a median progression-free survival of 8.3 months and an overall survival of 22.8 months. The drug works by overcoming resistance mechanisms to tyrosine kinase inhibitors, targeting MET as a bypass resistance pathway and engaging effector cells for an anti-cancer effect. Real-world data comparisons suggest that amivantamab offers better outcomes than other therapies [141].

GB263T is a novel trispecific antibody targeting EGFR and MET, utilizing two humanized VHH antibodies that bind to distinct MET epitopes. The first VHH binding induces a conformational change that reveals the second epitope for sequential binding, enhancing tumor specificity and improving safety. In vitro studies show that GB263T promotes superior antigen-antibody endocytosis, more effectively blocks signal transduction pathways, and exhibits stronger ADCC activity compared to a benchmark antibody. Additionally, GB263T has shown promising preclinical efficacy in models with various EGFR/MET alterations, as the activation of EGFR- and MET-mediated signaling pathways is blocked [142].

### 4.4. MET-Targeted Antibody-Drug Conjugates

In addition to antibody-based therapies, antibody-drug conjugates (ADCs) are being developed to target MET. ADCs combine the specificity of monoclonal antibodies with the cytotoxic potency of chemotherapeutic agents. The efficacy of an ADC greatly relies on its intracellular trafficking and processing of its components to induce tumor cell death [143]. The concept behind ADCs is to selectively deliver toxic drugs directly to cancer cells, sparing healthy tissues and minimizing systemic side effects. Thus, MET-targeted ADCs can deliver cytotoxic agents directly to MET-expressing tumor cells, regardless of reliance on MET signaling. Telisotuzumab vedotin (ABBV-399) consists of a humanized monoclonal antibody, telisotuzumab, coupled to an anti-microtubule drug through a linker that is cleavable once inside the cancer cell [144]. It has demonstrated efficacy in NSCLC patients exhibiting MET overexpression [145]. REGN5093-M114 is a MET-MET biparatopic antibody linked to a cytotoxic inhibitor of microtubule assembly [146]. This compound is being explored in a phase I/II trial in MET-positive NSCLC. TR1801-ADC is a toxin-conjugated non agonistic anti-MET monoclonal antibody that can target tumors with even low MET expression [147]. Currently, TR1801-ADC is being assessed in a phase I clinical trial for MET-overexpressing solid tumors. Other ADCs, such as SHR-A1403 and MYTX-011, have been developed and demonstrated enhanced efficacy and safety compared to existing small-molecule inhibitors or monoclonal antibodies targeting MET-overexpressing cancers [148,149]. These newer ADCs show promise in delivering more precise and potent therapeutic effects, reducing off-target toxicity, and overcoming some limitations faced by earlier therapies.

### 4.5. Targeting the HGF/MET Axis in Tumor Microenvironment

The interaction between stromal HGF and MET-expressing cancer cells plays a crucial role in tumor development within the tumor microenvironment (TME). Activation of wild-type MET contributes to drug resistance, not only due to receptor overexpression but also as a result of paracrine HGF secretion by tumor-associated stromal cells. The tumor stroma is rich in proteases that can convert the inactive, single-chain form of pro-HGF into its active, two-chain form, which can then activate the MET receptor [21]. This abundant availability of the active ligand within the tumor’s interstitial space, combined with the elevated expression of MET receptors on cancer cells, may serve as a common mechanism by which tumors resist the effects of targeted therapies. This adaptive strategy is particularly pronounced in tumors with hypoxic or inflammatory characteristics, making it harder for treatments to remain effective over time. Cancer-associated fibroblasts (CAFs) are key producers of HGF, which signals to MET-expressing cancer cells, promoting their growth. Additionally, neutrophils and macrophages in the TME also contribute to HGF production, further supporting tumor growth through MET activation. HGF-neutralizing antibodies are potential therapeutic tools in disrupting this cross-communication signal between stromal and cancer cells.

Rilotumumab and ficlatuzumab are monoclonal antibodies targeting HGF. Rilotumumab (AMG 102) is a fully human monoclonal antibody that specifically targets HGF, preventing it from binding to the MET receptor. It was initially developed for several solid tumors, including gastric cancer [150,151,152]. In early trials, it showed promise when combined with chemotherapy, leading to improved progression-free survival in MET-positive patients [150]. However, its development was halted after phase III trials in advanced gastric cancer showed increased mortality when used with chemotherapy compared to chemotherapy alone, leading to the discontinuation of further studies in this area [153]. Neutralization of HGF-dependent survival cues might be one explanation for why HGF antibodies resulted in paradoxical detrimental outcomes. Ficlatuzumab (AV-299) is a humanized monoclonal antibody designed to neutralize HGF and inhibit the MET receptor. It has been evaluated in NSCLC in combination with EGFR inhibitors such as gefitinib and cetuximab [154,155]. While phase II trials in unselected populations did not show significant improvements in overall response rate or progression-free survival, certain subsets of patients with low MET levels or high stromal HGF exhibited better outcomes. Ficlatuzumab continues to be explored in clinical trials, showing potential in specific patient groups.

### 4.6. Current Limitations and Challenges

MET kinase inhibitors and MET-targeting monoclonal antibodies each offer distinct therapeutic benefits and limitations in treating MET-dependent cancers. The effectiveness of MET kinase inhibitors is often limited by resistance mechanisms such as mutations that bypass MET inhibition or tumor microenvironment factors that reduce inhibitor efficacy. Monoclonal antibodies focus on the extracellular domain of the MET receptor, blocking ligand binding and/or promoting receptor degradation. Although this can overcome some resistance issues, clinical responses have been inconsistent. The variable efficacy of antibody-based approaches arises partly because antibodies may not sufficiently penetrate solid tumors or effectively induce immune-mediated tumor destruction. Additionally, some tumor types or subtypes may have adaptive responses that reduce sensitivity to antibody-based therapies. Newer antibody formulations, like SYM015, which targets multiple MET epitopes, or amivantamab, which targets both the MET and EGFR pathways, are showing promise by engaging immune mechanisms that enhance tumor destruction or offering a way to address resistance mechanisms that often arise with single-target therapies. Overall, combining TKIs with monoclonal antibodies or other targeted treatments may offer improved results by addressing diverse resistance mechanisms. Challenges persist in refining delivery systems and overcoming resistance mechanisms, highlighting the critical need for precise molecularly targeted therapies. Advances in protein degradation techniques and enhancing ADC efficacy are essential to optimize therapeutic impact and mitigate resistance pathways, underscoring the importance of continued research in these areas.

## 5. Conclusions

In the context of cancer, the MET oncogene contributes to tumorigenesis, metastasis, and therapeutic resistance, positioning the HGF/MET axis as a significant target for the development of novel anti-cancer therapies. The growing understanding of MET signaling has facilitated the development of innovative therapeutic strategies targeting MET. These approaches, ranging from small-molecule kinase inhibitors, degraders, and monoclonal antibodies to advanced drug delivery systems, highlight the potential for personalized and effective cancer treatments. Ongoing research and clinical trials will continue to refine these applications, aiming to improve patient outcomes in MET-driven cancers.

## 6. Methodology 

PubMed and Google were the primary databases used to search for the cited references. Targeted search terms included “MET receptor”, “HGF ligand”, “MET inhibitors”, “MET small molecule degrader”, “MET/HGF-targeted antibodies”, and “MET-targeted antibody-drug conjugates”. The selected references comprised studies on MET signaling mechanisms, clinical and preclinical evaluations of MET inhibitors, and emerging drug strategies. The review emphasized high-quality, peer-reviewed articles, clinical trial data, and foundational studies on MET-driven cancers. Reviews or articles that did not present new or original data were excluded. In addition, updated references published in recent years were prioritized. Selected articles were downloaded in Mendeley Library and organized according to the type of therapeutic strategy. The scope of this review, focused on a practical synthesis, inherently limits inclusion of all available literature.

## Figures and Tables

**Table 1:** MET-targeting drugs.

HGF/MET Inhibitor	Strategy	Target	Tumor	Approval Status/ Clinical Research Progress	Reference
Tepotinib	TKI	MET	NSCLC, advanced HCC	US FDA approval for metastatic NSCLC with MET exon 14 skipping mutations (2021)	[65,66,67,68]
Capmatinib	TKI	MET	NSCLC	US FDA approval for metastatic NSCLC with MET exon 14 skipping mutations (2020)	[68,69,70]
Savolitinib	TKI	MET	NSCLC, advanced RCC	China approval for metastatic NSCLC with MET exon 14 skipping mutations (2021)	[71,72,73,74,75]
Bozitinib	TKI	MET	Advanced NSCLC, glioblastoma	Phase II trial study for metastatic NSCLC with MET exon 14 skipping mutations (2024)	[76,77]
Crizotinib	TKI	MET/ALK	RCC, NSCLC, sALCL	US FDA approval for metastatic ALK-positive NSCLC (2011) and refractory sALCL (2021)	[78,79,80,81,82,83]
Cabozantinib	TKI	MET/VEGFR2	Metastatic CRC, RCC, pancreatic cancer, hepatocellular carcinoma, neuroblastoma, thyroid cancer	US FDA approval for advanced RCC (2016), hepatocellular carcinoma (2019), thyroid cancer (2021)	[84,85,86,87,88,89]
Foretinib	TKI	MET/VEGFR2	NSCLC, HNC, RCC, metastatic GC, metastatic BC, advanced HCC	Phase II trial study for HNC (2012), RCC (2012), metastatic GC (2013), metastatic BC (2016), advanced HCC (2017)	[90,91,92,93,94,95,96,97]
Golvatinib	TKI	MET/VEGFR	NSCLC, advanced solid tumors	Phase I trial study for advanced solid tumors (2014)	[90,91,92,98]
Merestinib	TKI	MET/AXL	NSCLC, Advanced solid tumors	Phase I trial study for advanced solid tumors (2019), phase II trial study for NSCLC (ongoing)	[90,91,92,99]
Glesatinib	TKI	MET/SMO	NSCLC, advanced solid tumors	Phase II trial study for NSCLC (2024)	[90,91,92,100]
Onartuzumab	mAb	MET	NSCLC, metastatic CRC, gastroesophageal adenocarcinoma	Phase III trial study for NSCLC (2016), gastroesophageal adenocarcinoma (2017), phase II trial study for metastatic CRC (2017)	[101,102,103,104]
Emibetuzumab	mAb	MET	MET-addicted cancer, NSCLC	Phase II trial study for NSCLC (2020)	[105,106]
DN30	mAb	MET	MET-addicted cancer	Preclinical studies	[107,108,109,110]
SYM015	mAb	MET	MET-addicted cancer	Preclinical studies	[111,112]
REGN5093	mAb	MET	MET-addicted cancer	Preclinical studies	[113]
Amivantamab	mAb	MET/EGFR	NSCLC	US FDA approval as EGFR drug inhibitor for NSCLC (2021)	[114,115,116,117,118,119]
GB263T	mAb	MET/EGFR	NSCLC	Preclinical studies	[120]
ABBV-399	ADC	MET mAb + anti-microtubule drug	MET-addicted cancer, NSCLC	Phase II trial study for NSCLC (2024)	[121,122,123]
REGN5093-M114	ADC	MET mAb + cytotoxic microtubule assembly inhibitor	NSCLC	Preclinical studies	[124]
TR1801-ADC	ADC	MET mAb + toxin	MET-addicted cancer	Preclinical studies	[125]
SHR-A1403	ADC	MET mAb + toxin	MET-addicted cancer	Preclinical studies	[126]
MYTX-011	ADC	MET mAb + toxin	MET-addicted cancer	Preclinical studies	[127]
Rilotumumab	mAb	HGF	Metastatic RCC, glioblastoma, GC	Phase III trial study for GC (2017), Phase II trial study for metastatic RCC and glioblastoma (2011)	[128,129,130,131]
Ficlatuzumab	mAb	HGF	Metastatic HNC, NSCLC	Phase II trial study for metastatic HNC (2023), phase I trial study for NSCLC (2018)	[132,133]

ADC: antibody-drug conjugate; BC: breast cancer; CRC: colorectal cancer; HCC: hepatocellular carcinoma; HNC: head and neck cancer; GC: gastric cancer; mAb: monoclonal antibody; NSCLC: non-small-cell lung cancer; RCC: renal cell carcinoma; sALCL: systemic anaplastic large cell lymphoma; TKI: tyrosine kinase inhibitor; TNBC: triple negative breast cancer; US FDA: United States Food and Drug Administration.

## Data Availability

No new data were created or analyzed in this study. Data sharing is not applicable to this article.

## Abbreviations

ADCs: antibody-drug conjugates; CAFs: cancer-associated fibroblasts; EMT: epithelial to mesenchymal transition; Exon20ins: Exon20 insertions; GGA3: Golgi-localized γ-ear-containing Arf-binding protein 3; HGF: hepatocyte growth factor; HyT: hydrophobic tags; LRIG1: immunoglobulin-like domain-containing protein 1; NSCLC: non-small-cell lung cancer; PAK: p21-activated kinase; PROTACs: proteolysis-targeting chimeras; RCP: Rab coupling protein; RTK: receptor tyrosine kinase; SEMA domain: semaphoring-like domain; SF: scatter factor; sMET: soluble decoy form of MET; TME: tumor microenvironment; TNBC: triple negative breast cancer; TPD: targeted protein degradation; TPR: translocated promoter region.

## References

[1] Nakamura T., Nawa K., Ichihara A. (1984). Partial purification and characterization of hepatocyte growth factor from serum of hepatectomized rats. Biochem. Biophys. Res. Commun..

[2] Naldini L., Weidner K.M., Vigna E., Gaudino G., Bardelli A., Ponzetto C., Narsimhan R.P., Hartmann G., Zarnegar R., Michalopoulos G.K. (1991). Scatter factor and hepatocyte growth factor are indistinguishable ligands for the MET receptor. Embo J..

[3] Birchmeier C., Birchmeier W., Gherardi E., Vande Woude G.F. (2003). Met, metastasis, motility and more. Nat. Rev. Mol. Cell Biol..

[4] Trusolino L., Bertotti A., Comoglio P.M. (2010). MET signalling: Principles and functions in development, organ regeneration and cancer. Nat. Rev. Mol. Cell Biol..

[5] Birchmeier C., Gherardi E. (1998). Developmental roles of HGF/SF and its receptor, the c-met tyrosine kinase. Trends Cell Biol..

[6] Desole C., Gallo S., Vitacolonna A., Montarolo F., Bertolotto A., Vivien D., Comoglio P., Crepaldi T. (2021). HGF and MET: From brain development to neurological disorders. Front. Cell Dev. Biol..

[7] Bladt F., Riethmacher D., Isenmann S., Aguzzi A., Birchmeier C. (1995). Essential role for the c-met receptor in the migration of myogenic precursor cells into the limb bud. Nature.

[8] Uehara Y., Minowa O., Mori C., Shiota K., Kuno J., Noda T., Kitamura N. (1995). Placental defect and embryonic lethality in mice lacking hepatocyte growth factor/scatter factor. Nature.

[9] Maina F., Casagranda F., Audero E., Simeone A., Comoglio P.M., Klein R., Ponzetto C. (1996). Uncoupling of Grb2 from the Met receptor in vivo reveals complex roles in muscle development. Cell.

[10] Maina F., Klein R. (1999). Hepatocyte growth factor, a versatile signal for developing neurons. Nat. Neurosci..

[11] Schmidt C., Bladt F., Goedecke S., Brinkmann V., Zschiesche W., Sharpe M., Gherardi E., Birchmeier C. (1995). Scatter factor/hepatocyte growth factor is essential for liver development. Nature.

[12] Bryant D.M., Mostov K.E. (2008). From cells to organs: Building polarized tissue. Nature Rev. Mol. Cell Biol..

[13] Montesano R., Schaller G., Orci L. (1991). Induction of epithelial tubular morphogenesis in vitro by fibroblast-derived soluble factors. Cell.

[14] Bussolino F., Di Renzo M.F., Ziche M., Bocchietto E., Olivero M., Naldini L., Gaudino G., Tamagnone L., Coffer A., Comoglio P.M. (1992). Hepatocyte growth factor is a potent angiogenic factor which stimulates endothelial cell motility and growth. J. Cell Biol..

[15] Boccaccio C., Andò M., Tamagnone L., Bardelli A., Michieli P., Battistini C., Comoglio P.M. (1998). Induction of epithelial tubules by growth factor HGF depends on the STAT pathway. Nature.

[16] Müller M., Morotti A., Ponzetto C. (2002). Activation of NF-κB is essential for hepatocyte growth factormediated proliferation and tubulogenesis. Mol. Cell Biol..

[17] Liu Y. (2004). Hepatocyte growth factor in kidney fibrosis: Therapeutic potential and mechanisms of action. Am. J. Physiol. Renal Physiol..

[18] Gurtner G.C., Werner S., Barrandon Y., Longaker M.T. (2008). Wound repair and regeneration. Nature.

[19] Blanpain C., Fuchs E. (2009). Epidermal homeostasis: A balancing act of stem cells in the skin. Nature Rev. Mol. Cell Biol..

[20] Chmielowiec J., Borowiak M., Morkel M., Stradal T., Munz B., Werner S., Wehland J., Birchmeier C., Birchmeier W. (2007). c-Met is essential for wound healing in the skin. J. Cell Biol..

[21] Comoglio P., Trusolino L., Boccaccio C. (2018). Known and novel roles of the MET oncogene in cancer: A coherent approach to targeted therapy. Nat. Rev. Cancer.

[22] Trusolino L., Comoglio P.M. (2002). Scatter-factor and semaphorin receptors: Cell signalling for invasive growth. Nature Rev. Cancer.

[23] Lai A.Z., Abella J.V., Park M. (2009). Crosstalk in Met receptor oncogenesis. Trends Cell Biol..

[24] Boccaccio C., Comoglio P.M. (2006). Invasive growth: A MET-driven genetic programme for cancer and stem cells. Nat. Rev. Cancer.

[25] Yang J., Weinberg R.A. (2008). Epithelial-mesenchymal transition: At the crossroads of development and tumor metastasis. Dev. Cell.

[26] Ye X., Weinberg R.A. (2015). Epithelial-mesenchymal plasticity: A central regulator of cancer progression. Trends Cell Biol..

[27] Montagner A., Yart A., Dance M., Perret B., Salles J.P., Raynal P. (2005). A novel role for Gab1 and SHP2 in epidermal growth factor-induced Ras activation. J. Biol. Chem..

[28] Baldanzi G., Graziani A. (2015). Physiological Signaling and Structure of the HGF Receptor MET. Biomedicines.

[29] Altintas D.M., Comoglio P.M. (2023). An observatory for the MET oncogene: A guide for targeted therapies. Cancers.

[30] Comoglio P.M., Giordano S., Trusolino L. (2008). Drug development of MET inhibitors: Targeting oncogene addiction and expedience. Nat. Rev. Drug Discov..

[31] Gherardi E., Birchmeier W., Birchmeier C., Vande W.G. (2012). Targeting MET in cancer: Rationale and progress. Nat. Rev. Cancer.

[32] Turke A.B., Zejnullahu K., Wu Y.L., Song Y., Dias-Santagata D., Lifshits E., Toschi L., Rogers A., Mok T., Sequist L. (2010). Preexistence and clonal selection of MET amplification in EGFR mutant NSCLC. Cancer Cell.

[33] Bardelli A., Corso S., Bertotti A., Hobor S., Valtorta E., Siravegna G., Sartore-Bianchi A., Scala E., Cassingena A., Zecchin D. (2013). Amplification of the MET receptor drives resistance to anti-EGFR therapies in colorectal cancer. Cancer Discov..

[34] Pietrantonio F., Oddo D., Gloghini A., Valtorta E., Berenato R., Barault L., Caporale M., Busico A., Morano F., Gualeni A.V. (2016). MET-Driven Resistance to Dual EGFR and BRAF Blockade May Be Overcome by Switching from EGFR to MET Inhibition in BRAF-Mutated Colorectal Cancer. Cancer Discov..

[35] Schmidt L., Duh F.M., Chen F., Kishida T., Glenn G., Choyke P., Scherer S.W., Zhuang Z., Lubensky I., Dean M. (1997). Germline and somatic mutations in the tyrosine kinase domain of the MET proto-oncogene in papillary renal carcinomas. Nat. Genet..

[36] Graveel C.R., London C.A., Woude G.F.V. (2005). A mouse model of activating Met mutations. Cell Cycle.

[37] Frampton G.M., Ali S.M., Rosenzweig M., Chmielecki J., Lu X., Bauer T.M., Akimov M., Bufill J.A., Lee C., Jentz D. (2015). Activation of MET via diverse exon 14 splicing alterations occurs in multiple tumor types and confers clinical sensitivity to MET inhibitors. Cancer Discov..

[38] Tong J.H., Yeung S.F., Chan A.W., Chung L.Y., Chau S.L., Lung R.W., Tong C.Y., Chow C., Tin E.K., Yu Y.H. (2016). MET Amplification and Exon 14 Splice Site Mutation Define Unique Molecular Subgroups of Non-Small Cell Lung Carcinoma with Poor Prognosis. Clin. Cancer Res..

[39] Liu S.Y., Gou L.Y., Li A.N., Lou N.N., Gao H.F., Su J., Yang J.J., Zhang X.C., Shao Y., Dong Z.Y. (2016). The unique characteristics of MET Exon 14 mutation in Chinese patients with NSCLC. J. Thorac. Oncol..

[40] Vuong H.G., Ho A.T.N., Altibi A.M.A., Nakazawa T., Katoh R., Kondo T. (2018). Clinicopathological implications of MET exon 14 mutations in non-small cell lung cancer—A systematic review and meta-analysis. Lung Cancer.

[41] Soman N.R., Correa P., Ruiz B.A., Wogan G.N. (1991). The TPR-MET oncogenic rearrangement is present and expressed in human gastric carcinoma and precursor lesions. Proc. Natl. Acad. Sci. USA.

[42] Liang T.J., Reid A.E., Xavier R., Cardiff R.D., Wang T.C. (1996). Transgenic expression of tpr-met oncogene leads to development of mammary hyperplasia and tumors. J. Clin. Investig..

[43] International Cancer Genome Consortium PedBrain Tumor Project (2016). Recurrent MET fusion genes represent a drug target in pediatric glioblastoma. Nat. Med..

[44] Ivan M., Bond J.A., Prat M., Comoglio P.M., Winford-Thomas D. (1997). Activated ras and ret oncogenes induce over-expression of c-met (hepatocyte growth factor receptor) in human thyroid epithelial cells. Oncogene.

[45] Pennacchietti S., Michieli P., Galluzzo M., Mazzone M., Giordano S., Comoglio P.M. (2003). Hypoxia promotes invasive growth by transcriptional activation of the met protooncogene. Cancer Cell..

[46] Boccaccio C., Gaudino G., Gambarotta G., Galimi F., Comoglio P.M. (1994). Hepatocyte growth factor (HGF) receptor expression is inducible and is part of the delayed-early response to HGF. J. Biol. Chem..

[47] Bhowmick N.A., Neilson E.G., Moses H.L. (2004). Stromal fibroblasts in cancer initiation and progression. Nature.

[48] Ponzetto C., Zhen Z., Audero E., Maina F., Bardelli A., Basile M.L., Giordano S., Narsimhan R., Comoglio P. (1996). Specific uncoupling of GRB2 from the Met receptor. Differential effects on transformation and motility. J. Biol. Chem..

[49] Koch J.P., Aebersold D.M., Zimmer Y., Medová M. (2020). MET targeting: Time for a rematch. Oncogene.

[50] Hammond D.E., Urbe S., vande Woude G.F., Clague M.J. (2001). Down-regulation of Met, the receptor for hepatocyte growth factor. Oncogene.

[51] Petrelli A., Gilestro G.F., Lanzardo S., Comoglio P.M., Migone N., Giordano S. (2002). The endophilin–CIN85–CBL complex mediates ligand-dependent downregulation of c-Met. Nature.

[52] Li N., Lorinczi M., Ireton K., Elferink L.A. (2007). Specific Grb2-mediated interactions regulate clathrin-dependent endocytosis of the c-Met-tyrosine kinase. J. Biol. Chem..

[53] Onozato R., Kosaka T., Kuwano H., Sekido Y., Yatabe Y., Mitsudomi T. (2009). Activation of MET by gene amplification or by splice mutations deleting the juxtamembrane domain in primary resected lung cancers. J. Thorac. Oncol..

[54] Hammond D.E., Carter S., McCullough J., Urbe S., vande Woude G., Clague M.J. (2003). Endosomal dynamics of met determine signaling output. Mol. Biol. Cell.

[55] Muharram G., Sahgal P., Korpela T., de Franceschi N., Kaukonen R., Clark K., Tulasne D., Carpen O., Ivaska J. (2014). Tensin-4-dependent met stabilization is essential for survival and proliferation in carcinoma cells. Dev. Cell.

[56] Muller P.A., Trinidad A.G., Timpson P., Morton J.P., Zanivan S., van den Berghe P.V., Nixon C., Karim S.A., Caswell P.T., Noll J.E. (2013). Mutant p53 enhances met trafficking and signalling to drive cell scattering and invasion. Oncogene.

[57] Parachoniak C.A., Luo Y., Abella J.V., Keen J.H., Park M. (2011). Gga3 functions as a switch to promote met receptor recycling, essential for sustained erk and cell migration. Dev. Cell.

[58] Joffre C., Barrow R., Menard L., Calleja V., Hart I.R., Kermorgant S. (2011). A direct role for Met endocytosis in tumorigenesis. Nat. Cell Biol..

[59] Lefebvre J., Ancot F., Leroy C., Muharram G., Lemière A., Tulasne D. (2012). Met degradation: More than one stone to shoot a receptor down. FASEB J..

[60] Kopitz C., Gerg M., Bandapalli O.R., Ister D., Pennington C.J., Hauser S., Flechsig C., Krell H.W., Antolovic D., Brew K. (2007). Tissue inhibitor of metalloproteinases-1 promotes liver metastasis by induction of hepatocyte growth factor signaling. Cancer Res..

[61] Foveau B., Ancot F., Leroy C., Petrelli A., Reiss K., Vingtdeux V., Giordano S., Fafeur V., Tulasne D. (2009). Down-regulation of the met receptor tyrosine kinase by presenilin-dependent regulated intramembrane proteolysis. Mol. Biol. Cell.

[62] Sangwan V., Paliouras G.N., Abella J.V., Dubé N., Monast A., Tremblay M.L., Park M. (2008). Regulation of the Met receptor-tyrosine kinase by the protein-tyrosine phosphatase 1B and T-cell phosphatase. J. Biol. Chem..

[63] Kermorgant S., Parker P.J. (2008). Receptor trafficking controls weak signal delivery: A strategy used by c-met for stat3 nuclear accumulation. J. Cell Biol..

[64] Menard L., Parker P.J., Kermorgant S. (2014). Receptor tyrosine kinase c-Met controls the cytoskeleton from different endosomes via different pathways. Nat. Commun..

[65] Crepaldi T., Gallo S., Comoglio P.M. (2024). The MET Oncogene: Thirty years of insights into molecular mechanisms driving malignancy. Pharmaceuticals.

[66] Paik P.K., Felip E., Veillon R., Sakai H., Cortot A.B., Garassino M.C., Mazieres J., Viteri S., Senellart H., Van Meerbeeck J. (2020). Tepotinib in non-small-cell lung cancer with MET exon 14 skipping mutations. N. Engl. J. Med..

[67] Choueiri T.K., Escudier B., Powles T., Tannir N.M., Mainwaring P.N., Rini B.I., Hammers H.J., Donskov F., Roth B.J., Peltola K. (2016). Cabozantinib versus everolimus in advanced renal cell carcinoma (METEOR): Final results from a randomised, open-label, phase 3 trial. Lancet Oncol..

[68] Mathieu L.N., Larkins E., Akinboro O., Roy P., Amatya A.K., Fiero M.H., Mishra-Kalyani P.S., Helms W.S., Myers C.E., Skinner A.M. (2022). FDA Approval Summary: Capmatinib and Tepotinib for the treatment of metastatic NSCLC harboring MET Exon 14 Skipping mutations or alterations. Clin. Cancer Res..

[69] Mazieres J., Paik P.K., Garassino M.C., Le X., Sakai H., Veillon R., Smit E.F., Cortot A.B., Raskin J., Viteri S. (2023). Tepotinib treatment in patients with MET exon 14-skipping non-small cell lung cancer: Long-term follow-up of the VISION Phase 2 nonrandomized clinical trial. JAMA Oncol..

[70] Wolf J., Seto T., Han J.Y., Reguart N., Garon E.B., Groen H.J.M., Tan D.S.W., Hida T., de Jonge M., Orlov S.V. (2020). Capmatinib in MET exon 14-mutated or MET-amplified non-small-cell lung cancer. N. Engl. J. Med..

[71] Wu Y.L., Smit E.F., Bauer T.M. (2021). Capmatinib for patients with non-small cell lung cancer with MET exon 14 skipping mutations: A review of preclinical and clinical studies. Cancer Treat. Rev..

[72] Sennino B., Ishiguro-Oonuma T., Wei Y., Naylor R.M., Williamson C.W., Bhagwandin V., Tabruyn S.P., You W.K., Chapman H.A., Christensen J.G. (2012). Suppression of tumor invasion and metastasis by concurrent inhibition of c-Met and VEGF signaling in pancreatic neuroendocrine tumors. Cancer Discov..

[73] Daudigeos-Dubus E., Le Dret L., Bawa O., Opolon P., Vievard A., Villa I., Bosq J., Vassal G., Geoerger B. (2017). Dual inhibition using cabozantinib overcomes HGF/MET signaling mediated resistance to pan-VEGFR inhibition in orthotopic and metastatic neuroblastoma tumors. Int. J. Oncol..

[74] Elisei R., Schlumberger M.J., Müller S.P., Schöffski P., Brose M.S., Shah M.H., Licitra L., Jarzab B., Medvedev V., Kreissl M.C. (2013). Cabozantinib in progressive medullary thyroid cancer. J. Clin. Oncol..

[75] Markham A. (2021). Savolitinib: First Approval. Drugs.

[76] Lu S., Fang J., Li X., Cao L., Zhou J., Guo Q., Liang Z., Cheng Y., Jiang L., Yang N. (2021). Once-daily savolitinib in Chinese patients with pulmonary sarcomatoid carcinomas and other non-small-cell lung cancers harbouring MET exon 14 skipping alterations: A multicentre, single-arm, open-label, phase 2 study. Lancet Respir. Med..

[77] Lefler D.S., Tierno M.B., Bashir B. (2022). Partial treatment response to capmatinib in MET-amplified metastatic intrahepatic cholangiocarcinoma: Case report & review of literature. Cancer Biol. Ther..

[78] Turpin A., Descarpentries C., Grégoire V., Farchi O., Cortot A.B., Jamme P. (2023). Response to Capmatinib in a MET Fusion-positive Cholangiocarcinoma. Oncologist.

[79] Li R., Liu X., Xu Y., Zhao J., Zhong W., Gao X., Chen M., Wang M. (2024). Remarkable pathological response to neoadjuvant tepotinib in lung adenocarcinoma with MET exon 14 skipping mutation: A case report. Thorac. Cancer.

[80] Jóri B., Bundschuh O., Falk M., Heukamp L.C., Kluge A., Tiemann M., Willborn K.C., Woitzik J., Griesinger F. (2024). Intracranial response to capmatinib after progression on crizotinib in a patient with MET exon 14 skipping non-small cell lung cancer—A case report. Transl. Lung Cancer Res..

[81] Scott A.J., Basu Mallick A., Dotan E., Cohen S.J., Gold P.J., Hochster H.S., Subramaniam S., Barzi A., Watts G.S., Blatchford P.J. (2022). A Phase II study investigating cabozantinib in patients with refractory metastatic colorectal cancer (AGICC 17CRC01). Cancer Res. Commun..

[82] Kazandjian D., Blumenthal G.M., Chen H.Y., He K., Patel M., Justice R., Keegan P., Pazdur R. (2014). FDA approval summary: Crizotinib for the treatment of metastatic non-small cell lung cancer with anaplastic lymphoma kinase rearrangements. Oncologist.

[83] Merino M., Kasamon Y., Li H., Ma L., Leong R., Zhou J., Reaman G., Chambers W., Richardson N., Theoret M. (2022). FDA approval summary: Crizotinib for pediatric and young adult patients with relapsed or refractory systemic anaplastic large cell lymphoma. Pediatr. Blood Cancer.

[84] Tian J., Lin Z., Chen Y., Fu Y., Ding Z. (2022). Dramatic response to neoadjuvant savolitinib in marginally resectable lung adenocarcinoma with MET exon 14 skipping mutation: A case report and literature review. Front. Oncol..

[85] Hu H., Mu Q., Bao Z., Chen Y., Liu Y., Chen J., Wang K., Wang Z., Nam Y., Jiang B. (2018). Mutational landscape of secondary glioblastoma guides MET-targeted trial in brain tumor. Cell.

[86] Yang J.J., Zhang Y., Wu L., Hu J., Wang Z.H., Chen J.H., Fan Y., Lin G., Wang Q.M., Yao Y. (2024). Vebreltinib for advanced non-small cell lung cancer harboring c-Met Exon 14 skipping mutation: A multicenter, single-arm, phase II KUNPENG study. J. Clin. Oncol..

[87] Huang S., Li L., Yan N., Zhang H., Guo Q., Guo S., Geng D., Liu X., Li X. (2024). Case report: The effect of second-line vebreltinib treatment on a patient with advanced NSCLC harboring the MET exon 14 skipping mutation after tepotinib treatment. Front. Oncol..

[88] Drilon A., Clark J.W., Weiss J., Ou S.I., Camidge D.R., Solomon B.J., Otterson G.A., Villaruz L.C., Riely G.J., Heist R.S. (2020). Antitumor activity of crizotinib in lung cancers harboring a MET exon 14 alteration. Nat. Med..

[89] Duke E.S., Barone A.K., Chatterjee S., Mishra-Kalyani P.S., Shen Y.L., Isikwei E., Zhao H., Bi Y., Liu J., Rahman N.A. (2022). FDA Approval Summary: Cabozantinib for Differentiated Thyroid Cancer. Clin. Cancer Res..

[90] Friedlaender A., Drilon A., Banna G.L., Peters S., Addeo A. (2020). The METeoric rise of MET in lung cancer. Cancer.

[91] Camidge D.R., Otterson G.A., Clark J.W., Ignatius Ou S.H., Weiss J., Ades S., Shapiro G.I., Socinski M.A., Murphy D.A., Conte U. (2021). Crizotinib in patients with MET-amplified NSCLC. J. Thorac. Oncol..

[92] Moro-Sibilot D., Cozic N., Pérol M., Mazières J., Otto J., Souquet P.J., Bahleda R., Wislez M., Zalcman G., Guibert S.D. (2019). Crizotinib in c-MET- or ROS1-positive NSCLC: Results of the AcSé phase II trial. Ann. Oncol..

[93] Seiwert T., Sarantopoulos J., Kallender H., McCallum S., Keer H.N., Blumenschein G. (2013). Phase II trial of single-agent foretinib (GSK1363089) in patients with recurrent or metastatic squamous cell carcinoma of the head and neck. Investig. New Drugs.

[94] Choueiri T.K., Vaishampayan U., Rosenberg J.E., Logan T.F., Harzstark A.L., Bukowski R.M., Rini B.I., Srinivas S., Stein M.N., Adams L.M. (2013). Phase II and biomarker study of the dual MET/VEGFR2 inhibitor foretinib in patients with papillary renal cell carcinoma. J. Clin. Oncol..

[95] Shah M.A., Wainberg Z.A., Catenacci D.V., Hochster H.S., Ford J., Kunz P., Lee F.C., Kallender H., Cecchi F., Rabe D.C. (2013). Phase II study evaluating 2 dosing schedules of oral foretinib (GSK1363089), cMET/VEGFR2 inhibitor, in patients with metastatic gastric cancer. PLoS ONE.

[96] Yau T.C.C., Lencioni R., Sukeepaisarnjaroen W., Chao Y., Yen C.J., Lausoontornsiri W., Chen P.J., Sanpajit T., Camp A., Cox D.S. (2017). A Phase I/II Multicenter Study of Single-Agent Foretinib as First-Line Therapy in Patients with Advanced Hepatocellular Carcinoma. Clin. Cancer Res..

[97] Rayson D., Lupichuk S., Potvin K., Dent S., Shenkier T., Dhesy-Thind S., Ellard S.L., Prady C., Salim M., Farmer P. (2016). Canadian Cancer Trials Group IND197: A phase II study of foretinib in patients with estrogen receptor, progesterone receptor, and human epidermal growth factor receptor 2-negative recurrent or metastatic breast cancer. Breast Cancer Res. Treat..

[98] Molife L.R., Dean E.J., Blanco-Codesido M., Krebs M.G., Brunetto A.T., Greystoke A.P., Daniele G., Lee L., Kuznetsov G., Myint K.T. (2014). A phase I, dose-escalation study of the multitargeted receptor tyrosine kinase inhibitor, golvatinib, in patients with advanced solid tumors. Clin. Cancer Res..

[99] He A.R., Cohen R.B., Denlinger C.S., Sama A., Birnbaum A., Hwang J., Sato T., Lewis N., Mynderse M., Niland M. (2019). First-in-Human Phase I Study of Merestinib, an Oral Multikinase Inhibitor, in Patients with Advanced Cancer. Oncologist.

[100] Hong D.S., Cappuzzo F., Chul Cho B., Dowlati A., Hussein M., Kim D.W., Percent I., Christensen J.G., Morin J., Potvin D. (2024). Phase II study investigating the efficacy and safety of glesatinib (MGCD265) in patients with advanced NSCLC containing MET activating alterations. Lung Cancer.

[101] Huang X., Li E., Shen H., Wang X., Tang T., Zhang X., Xu J., Tang Z., Guo C., Bai X. (2020). Targeting the HGF/MET Axis in Cancer Therapy: Challenges in Resistance and Opportunities for Improvement. Front. Cell Dev. Biol..

[102] Moosavi F., Giovannetti E., Peters G.J., Firuzi O. (2021). Combination of HGF/MET-targeting agents and other therapeutic strategies in cancer. Crit. Rev. Oncol. Hematol..

[103] Schöffski P., Wozniak A., Escudier B., Rutkowski P., Anthoney A., Bauer S., Sufliarsky J., van Herpen C., Lindner L.H., Grünwald V. (2017). Crizotinib achieves long-lasting disease control in advanced papillary renal-cell carcinoma type 1 patients with MET mutations or amplification. EORTC 90101 CREATE trial. Eur. J. Cancer.

[104] Bendell J.C., Hochster H., Hart L.L., Firdaus I., Mace J.R., McFarlane J.J., Kozloff M., Catenacci D., Hsu J.J., Hack S.P. (2017). A Phase II Randomized Trial (GO27827) of First-Line FOLFOX Plus Bevacizumab with or Without the MET Inhibitor Onartuzumab in Patients with Metastatic Colorectal Cancer. Oncologist.

[105] Choueiri T.K., Plimack E., Arkenau H.T., Jonasch E., Heng D.Y.C., Powles T., Frigault M.M., Clark E.A., Handzel A.A., Gardner H. (2017). Biomarker-based phase II trial of Savolitinib in patients with advanced papillary renal cell cancer. J. Clin. Oncol..

[106] Choueiri T.K., Heng D.Y.C., Lee J.L., Cancel M., Verheijen R.B., Mellemgaard A., Ottesen L.H., Frigault M.M., L’Hernault A., Szijgyarto Z. (2020). Efficacy of Savolitinib vs Sunitinib in patients with MET-driven papillary renal cell carcinoma: The SAVOIR phase 3 randomized clinical trial. JAMA Oncol..

[107] Ryoo B.Y., Cheng A.L., Ren Z., Kim T.Y., Pan H., Rau K.M., Choi H.J., Park J.W., Kim J.H., Yen C. (2021). Randomised Phase 1b/2 trial of tepotinib vs sorafenib in Asian patients with advanced hepatocellular carcinoma with MET overexpression. Br. J. Cancer.

[108] Al-Ghabkari A., Huang B., Park M. (2024). Aberrant MET receptor tyrosine kinase signaling in glioblastoma: Targeted therapy and future directions. Cells.

[109] Ying S., Chi H., Wu X., Zeng P., Chen J., Fu T., Fu W., Zhang P., Tan W. (2024). Selective and orally bioavailable c-Met PROTACs for the treatment of c-Met-addicted cancer. J. Med. Chem..

[110] Burslem G.M., Smith B.E., Lai A.C., Jaime-Figueroa S., McQuaid D.C., Bondeson D.P., Toure M., Dong H., Qian Y., Wang J. (2018). The advantages of targeted protein degradation over inhibition: An RTK case study. Cell Chem. Biol..

[111] Sachkova A.A., Andreeva D.V., Tikhomirov A.S., Scherbakov A.M., Salnikova D.I., Sorokin D.V., Bogdanov F.B., Rysina Y.D., Shchekotikhin A.E., Shchegravina E.S. (2022). Design, synthesis and in vitro investigation of Cabozantinib-Based PROTACs to target c-Met kinase. Pharmaceutics.

[112] Chen J.J., Jin J.M., Gu W.J., Zhao Z., Yuan H., Zhou Y.D., Nagle D.G., Xi Q.L., Zhang X.M., Sun Q.Y. (2023). Crizotinib-based proteolysis targeting chimera suppresses gastric cancer by promoting MET degradation. Cancer Sci..

[113] Békés M., Langley D.R., Crews C.M. (2022). PROTAC targeted protein degraders: The past is prologue. Nat. Rev. Drug Discov..

[114] Dale B., Cheng M., Park K.S., Kaniskan H.Ü., Xiong Y., Jin J. (2021). Advancing targeted protein degradation for cancer therapy. Nat. Rev. Cancer.

[115] Nishio M., Horiike A., Nokihara H., Horinouchi H., Nakamichi S., Wakui H., Ohyanagi F., Kudo K., Yanagitani N., Takahashi S. (2015). Phase I study of the anti-MET antibody onartuzumab in patients with solid tumors and MET-positive lung cancer. Investig. New Drugs.

[116] Spigel D.R., Edelman M.J., O’Byrne K., Paz-Ares L., Mocci S., Phan S., Shames D.S., Smith D., Yu W., Paton V.E. (2017). Results from the phase III randomized trial of onartuzumab plus erlotinib versus erlotinib in previously treated stage IIIB or IV non- small-cell lung cancer: METLung. J. Clin. Oncol..

[117] Shah M.A., Bang Y.J., Lordick F., Alsina M., Chen M., Hack S.P., Bruey J.M., Smith D., McCaffery I., Shames D.S. (2017). Effect of fluorouracil, leucovorin, and oxaliplatin with or without onartuzumab in HER2-negative, MET-positive gastroesophageal adenocarcinoma: The METGastric randomized clinical trial. JAMA Oncol..

[118] Moosavi F., Giovannetti E., Saso L., Firuzi O. (2019). HGF/MET pathway aberrations as diagnostic, prognostic, and predictive biomarkers in human cancers. Crit. Rev. Clin. Lab. Sci..

[119] Chon K., Larkins E., Chatterjee S., Mishra-Kalyani P.S., Aungst S., Wearne E., Subramaniam S., Li Y., Liu J., Sun J. (2023). FDA Approval Summary: Amivantamab for the Treatment of Patients with Non-Small Cell Lung Cancer with EGFR Exon 20 Insertion Mutations. Clin. Cancer Res..

[120] Liu L., Zeng W., Wortinger M.A., Yan S.B., Cornwell P., Peek V.L., Stephens J.R., Tetreault J.W., Xia J., Manro J.R. (2014). LY2875358, a neutralizing and internalizing anti-MET bivalent antibody, inhibits HGF-dependent and HGF-independent MET activation and tumor growth. Clin. Cancer Res..

[121] Prat M., Crepaldi T., Pennacchietti S., Bussolino F., Comoglio P.M. (1998). Agonistic monoclonal antibodies against the Met receptor dissect the biological responses to HGF. J. Cell Sci..

[122] Petrelli A., Circosta P., Granziero L., Mazzone M., Pisacane A., Fenoglio S., Comoglio P.M., Giordano S. (2006). Ab-induced ectodomain shedding mediates hepatocyte growth factor receptor down-regulation and hampers biological activity. Proc. Natl. Acad. Sci. USA.

[123] Camidge D.R., Bar J., Horinouchi H., Goldman J., Moiseenko F., Filippova E., Cicin I., Ciuleanu T., Daaboul N., Liu C. (2024). Telisotuzumab Vedotin Monotherapy in Patients With Previously Treated c-Met Protein-Overexpressing Advanced Nonsquamous *EGFR*-Wildtype Non-Small Cell Lung Cancer in the Phase II LUMINOSITY Trial. J. Clin. Oncol..

[124] Schelter F., Kobuch J., Moss M.L., Becherer J.D., Comoglio P.M., Boccaccio C., Krüger A. (2010). A disintegrin and metalloproteinase-10 (ADAM-10) mediates DN30 antibody-induced shedding of the Met surface receptor. J. Biol. Chem..

[125] Martinelli I., Modica C., Chiriaco C., Basilico C., Hughes J.M., Corso S., Giordano S., Comoglio P.M., Vigna E. (2022). hOA-DN30: A highly effective humanized single-arm MET antibody inducing remission of ‘MET-addicted’ cancers. J. Exp. Clin. Cancer Res..

[126] Modica C., Basilico C., Chiriaco C., Borrelli N., Comoglio P.M., Vigna E. (2021). A receptor-antibody hybrid hampering MET-driven metastatic spread. J. Exp. Clin. Cancer Res..

[127] Grandal M.M., Havrylov S., Poulsen T.T., Koefoed K., Dahlman A., Galler G.R., Conrotto P., Collins S., Eriksen K.W., Kaufman D. (2017). Simultaneous Targeting of Two Distinct Epitopes on MET Effectively Inhibits MET- and HGF-Driven Tumor Growth by Multiple Mechanisms. Mol. Cancer Ther..

[128] Poulsen T.T., Grandal M.M., Skartved N.J.Ø., Hald R., Alifrangis L., Koefoed K., Lindsted T., Fröhlich C., Pollmann S.E., Eriksen K.W. (2017). Sym015: A Highly Efficacious Antibody Mixture against MET-Amplified Tumors. Clin. Cancer Res..

[129] DaSilva J.O., Yang K., Perez Bay A.E., Andreev J., Ngoi P., Pyles E., Franklin M.C., Dudgeon D., Rafique A., Dore A. (2020). A biparatopic antibody that modulates MET trafficking exhibits enhanced efficacy compared with parental antibodies in MET-driven tumor models. Clin. Cancer Res..

[130] Moores S.L., Chiu M.L., Bushey B.S., Chevalier K., Luistro L., Dorn K., Brezski R.J., Haytko P., Kelly T., Wu S.J. (2016). A novel bispecific antibody targeting EGFR and cMet is effective against EGFR inhibitor-resistant lung tumors. Cancer Res..

[131] Vijayaraghavan S., Lipfert L., Chevalier K., Bushey B.S., Henley B., Lenhart R., Sendecki J., Beqiri M., Millar H.J., Packman K. (2020). Amivantamab (JNJ-61186372), an Fc enhanced EGFR/cMet bispecific antibody, induces receptor downmodulation and antitumor activity by monocyte/macrophage trogocytosis. Mol. Cancer Ther..

[132] Yun J., Lee S.H., Kim S.Y., Jeong S.Y., Kim J.H., Pyo K.H., Park C.W., Heo S.G., Yun M.R., Lim S. (2020). Antitumor activity of Amivantamab (JNJ-61186372), an EGFR-MET bispecific antibody, in diverse models of EGFR exon 20 insertion-driven NSCLC. Cancer Discov..

[133] Park K., Haura E.B., Leighl N.B., Mitchell P., Shu C.A., Girard N., Viteri S., Han J.Y., Kim S.W., Lee C.K. (2021). Amivantamab in EGFR exon 20 insertion-mutated non-small-cell lung cancer progressing on platinum chemotherapy: Initial results from the CHRYSALIS phase I study. J. Clin. Oncol..

[134] Lai G.G.Y., Guo R., Drilon A., Shao Weng Tan D. (2022). Refining patient selection of MET-activated non-small cell lung cancer through biomarker precision. Cancer Treat. Rev..

[135] Dong Y., Xu J., Sun B., Wang J., Wang Z. (2022). MET-targeted therapies and clinical outcomes: A systematic literature review. Mol. Diagn. Ther..

[136] Wang C., Lu X. (2023). Targeting MET: Discovery of Small Molecule Inhibitors as Non-Small Cell Lung Cancer Therapy. J. Med. Chem..

[137] Jin F., Lin Y., Yuan W., Wu S., Yang M., Ding S., Liu J., Chen Y. (2024). Recent advances in c-Met-based dual inhibitors in the treatment of cancers. Eur. J. Med. Chem..

[138] Suárez C., Larkin J.M.G., Patel P., Valderrama B.P., Rodriguez-Vida A., Glen H., Thistlethwaite F., Ralph C., Srinivasan G., Mendez-Vidal M.J. (2023). Phase II study investigating the safety and efficacy of savolitinib and durvalumab in metastatic papillary renal cancer (CALYPSO). J. Clin. Oncol..

[139] Min W., Yang H., Wang D., Chen C., Wang Y., Hou Y., Zhu Y., Sun C., Wang X., Yuan K. (2024). Discovery of potent and selective c-Met degraders for hepatocellular carcinoma treatment. J. Med. Chem..

[140] Scagliotti G., Moro-Sibilot D., Kollmeier J., Favaretto A., Cho E.K., Grosch H., Kimmich M., Girard N., Tsai C.M., Hsia T.C. (2020). A randomized-controlled phase II study of the MET antibody emibetuzumab in combination with erlotinib as first-line treatment for EGFR mutation-positive NSCLC patients. J. Thorac. Oncol..

[141] Minchom A., Viteri S., Bazhenova L., Gadgeel S.M., Ou S.I., Trigo J., Bauml J.M., Backenroth D., Bhattacharya A., Li T. (2022). Amivantamab compared with real-world therapies in patients with advanced non-small cell lung cancer harboring EGFR exon 20 insertion mutations who progressed after platinum-based chemotherapy. Lung Cancer.

[142] Zaman M., Huang C., Kankanamalage S.G., Chaudhary A.K., Dong J., Liu Y. (2021). Abstract LB069: Development of cMET/cMET/EGFR trispecific antibody as therapeutic modality for nonsmall cell lung cancer. Cancer Res..

[143] Chalouni C., Doll S. (2018). Fate of Antibody-Drug Conjugates in Cancer Cells. J. Exp. Clin. Cancer Res..

[144] Wang J., Anderson M.G., Oleksijew A., Vaidya K.S., Boghaert E.R., Tucker L., Zhang Q., Han E.K., Palma J.P., Naumovski L. (2017). ABBV-399, a c-Met antibody-drug conjugate that targets both MET-amplified and c-Met-overexpressing tumors, irrespective of MET pathway dependence. Clin. Cancer Res..

[145] Strickler J.H., Weekes C.D., Nemunaitis J., Ramanathan R.K., Heist R.S., Morgensztern D., Angevin E., Bauer T.M., Yue H., Motwani M. (2018). First-in-human phase I, dose-escalation and -expansion study of Telisotuzumab Vedotin, an antibody-drug conjugate targeting c-Met, in patients with advanced solid tumors. J. Clin. Oncol..

[146] Oh S.Y., Lee Y.W., Lee E.J., Kim J.H., Park Y., Heo S.G., Yu M.R., Hong M.H., DaSilva J., Daly C. (2023). Preclinical Study of a Biparatopic METxMET Antibody-Drug Conjugate, REGN5093-M114, Overcomes MET-driven Acquired Resistance to EGFR TKIs in EGFR-mutant NSCLC. Clin. Cancer Res..

[147] Gymnopoulos M., Betancourt O., Blot V., Fujita R., Galvan D., Lieuw V., Nguyen S., Snedden J., Stewart C., Villicana J. (2020). TR1801-ADC: A highly potent cMet antibody-drug conjugate with high activity in patient-derived xenograft models of solid tumors. Mol. Oncol..

[148] Yang C.Y., Wang L., Sun X., Tang M., Quan H.T., Zhang L.S., Lou L.G., Gou S.H. (2019). SHR-A1403, a novel c-Met antibody-drug conjugate, exerts encouraging anti-tumor activity in c-Met-overexpressing models. Acta Pharmacol. Sin..

[149] Gera N., Fitzgerald K.M., Ramesh V., Patel P., Kanojia D., Colombo F., Kien L., Aoyama S., Xu L., Jean J. (2024). MYTX-011: A pH-dependent anti-c-MET antibody-drug conjugate designed for enhanced payload delivery to c-MET-expressing tumor cells. Mol. Cancer Ther..

[150] Iveson T., Donehower R.C., Davidenko I., Tjulandin S., Deptala A., Harrison M., Nirni S., Lakshmaiah K., Thomas A., Jiang Y. (2014). Rilotumumab in combination with epirubicin, cisplatin, and capecitabine as first-line treatment for gastric or oesophagogastric junction adenocarcinoma: An open-label, dose de-escalation phase 1b study and a double-blind, randomised phase 2 study. Lancet Oncol..

[151] Wen P.Y., Schiff D., Cloughesy T.F., Raizer J.J., Laterra J., Smitt M., Wolf M., Oliner K.S., Anderson A., Zhu M. (2011). A phase II study evaluating the efficacy and safety of AMG 102 (rilotumumab) in patients with recurrent glioblastoma. Neuro Oncol..

[152] Schöffski P., Garcia J.A., Stadler W.M., Gil T., Jonasch E., Tagawa S.T., Smitt M., Yang X., Oliner K.S., Anderson A. (2011). A phase II study of the efficacy and safety of AMG 102 in patients with metastatic renal cell carcinoma. BJU Int..

[153] Catenacci D.V.T., Tebbutt N.C., Davidenko I., Murad A.M., Al-Batran S.E., Ilson D.H., Tjulandin S., Gotovkin E., Karaszewska B., Bondarenko I. (2017). Rilotumumab plus epirubicin, cisplatin, and capecitabine as first- line therapy in advanced MET- positive gastric or gastro- oesophageal junction cancer (RILOMET-1): A randomised, double- blind, placebo- controlled, phase 3 trial. Lancet Oncol..

[154] Tan E.H., Lim W.T., Ahn M.J., Ng Q.S., Ahn J.S., Shao-Weng Tan D., Sun J.M., Han M., Payumo F.C., McKee K. (2018). Phase 1b trial of Ficlatuzumab, a humanized hepatocyte growth factor inhibitory monoclonal antibody, in combination with Gefitinib in Asian patients with NSCLC. Clin. Pharmacol. Drug Dev..

[155] Bauman J.E., Saba N.F., Roe D., Bauman J.R., Kaczmar J., Bhatia A., Muzaffar J., Julian R., Wang S., Bearelly S. (2023). Randomized phase II trial of Ficlatuzumab with or without Cetuximab in pan-refractory, recurrent/metastatic head and neck cancer. J. Clin. Oncol..

